# Diabetes in pregnancy among First Nations women in Alberta, Canada: a retrospective analysis

**DOI:** 10.1186/1471-2393-14-136

**Published:** 2014-04-10

**Authors:** Richard T Oster, Malcolm King, Donald W Morrish, Maria J Mayan, Ellen L Toth

**Affiliations:** 1 Department of Medicine, University of Alberta, Edmonton, Alberta, Canada; 2 Faculty of Health Sciences, Simon Fraser University, Burnaby, British Columbia, Canada; 3 Faculty of Extension, University of Alberta, Edmonton, Alberta, Canada; 4 Department of Medicine, 4-030 Research Transition Facility, University of Alberta, Edmonton, Alberta T6G 2V2, Canada

**Keywords:** Indigenous population, Gestational diabetes, Pregnancy in diabetes, Epidemiology, Retrospective study

## Abstract

**Background:**

In addition to increasing the risk of adverse birth outcomes, diabetes in pregnancy is thought to be an important driver of the epidemic of type 2 diabetes affecting Canada’s First Nations population. The relative contributions of gestational diabetes mellitus (GDM) and pre-existing diabetes are not well understood. We generated a comprehensive epidemiological profile of diabetes in pregnancy over a 10-year period among the First Nations population of Alberta, Canada.

**Methods:**

De-identified administrative data for 427,058 delivery records were obtained for the years 2000–2009. Pregnancy risk factors and delivery outcomes were described and compared by ethnicity (First Nations vs. non-First Nations) and diabetes status. Age-adjusted prevalence values for GDM and pre-existing diabetes were calculated and were compared by ethnicity. Longitudinal changes over time were also examined. Predictors were explored using logistic regression analysis.

**Results:**

First Nations women had more antenatal risk factors and adverse infant outcomes that were compounded by diabetes. First Nations descent was an independent predictor of diabetes in pregnancy (p < 0.001). GDM prevalence was significantly higher among First Nations (6.1%) compared to non-First Nations women (3.8%; p < 0.001), but prevalence values increased significantly over time only in non-First Nations women (4.5 average annual percent change; p < 0.05). The prevalence of pre-existing diabetes was stable over time in both groups, but First Nations women experienced a 2.5-fold higher overall prevalence compared with non-First Nations women (1.5% vs. 0.6%, respectively; p < 0.001).

**Conclusions:**

Although First Nations women experience a higher overall prevalence of diabetes in pregnancy, the lack of increase in the prevalence over time is encouraging. However, because high-risk pregnancies and poor outcomes are more common among First Nations women, particularly those with diabetes, strategies to improve perinatal care must be implemented.

## Background

Diabetes in pregnancy [i.e., pre-existing diabetes and gestational diabetes mellitus (GDM)] is a major risk factor for perinatal complications, future obesity, and type 2 diabetes in mothers and their offspring [1-3]. This relationship is particularly strong if blood sugar remains unmanaged. Consequently, diabetes in pregnancy is considered to be a key predictor of, and contributor to, the current increases in type 2 diabetes worldwide, particularly in Indigenous populations [4]. Prevalence and incidence of type 2 diabetes has increased rapidly in Canada’s most populous Indigenous group (the First Nations) over the past half century and are currently 2–5 times greater than in the general Canadian population [5-7]. First Nations women generally suffer from a higher type 2 diabetes prevalence compared with their male counterparts [4,6-8].

Pregnancy may be a crucial point for interventions and appropriate healthcare aimed at reducing the prevalence of type 2 diabetes in First Nations peoples [2,9-11]. However, insufficient awareness and gaps in a detailed understanding of diabetes in pregnancy may be contributing to the lack of progress in reversing the epidemic. Knowledge about the extent of the problem of diabetes in pregnancy among First Nations women (namely prevalence, longitudinal trends in prevalence, and predictors of diabetes) is based primarily on cross-sectional studies focusing on crude GDM prevalence. Longitudinal data and data on pre-existing diabetes in pregnancy are limited. Other important maternal factors and pregnancy outcomes related to GDM and pre-existing diabetes in pregnancy have yet to be explored in this population. These factors and outcomes include the presence of pregestational hypertension, proteinuria or anemia, and smoking, alcohol consumption, illicit drug use, prior obstetrical history, breastfeeding, neonatal intensive care unit (NICU) admission, and preterm delivery. For these reasons, the current study was designed to use provincial administrative data to generate an epidemiological profile of First Nations diabetes in pregnancy in the province of Alberta.

## Methods

### Data

The Human Research Ethics Board of the University of Alberta approved the research protocol. Each province in Canada has different and separate procedures for collecting perinatal data. The Alberta Perinatal Health Program (APHP) comprehensively collects perinatal data from the provincial delivery record for all hospital births and registered midwife-attended home births. Delivery record information is recorded by a healthcare provider (usually a nurse) when a woman presents for delivery. This information is obtained from prenatal records and/or from the patient. Surveillance reports derived from Alberta delivery records and APHP data are also published [12]. Pertinent de-identified data from all delivery records were requested for the years 2000–2009 (complete data for 2010 and later were not available at the time of the request). Data from multiple pregnancies were included in the request. Data were complete or nearly complete (available for 97%–100% of pregnancies) for the majority of the variables.

During the time period of the obtained data, GDM diagnosis followed Canadian Diabetes Association guidelines [13]. Diagnosis included screening at 24–28 weeks with an initial 50-g oral glucose challenge and a subsequent blood glucose measurement at 1 hour post-consumption. A result of < 7.8 mmol L^-1^ was considered to be a normal result. A result of ≥ 10.3 mmol L^-1^ was considered to indicate the presence of GDM. A subsequent 75-g oral glucose tolerance test for the potential diagnosis of GDM was indicated to follow up a result between 7.8–10.2 mmol L^-1^. Two separate values of either fasting glucose ≥ 5.3 mmol L^-1^, a 1-hour post-consumption glucose ≥ 10.6 mmol L^-1^, or a 2-hour post-consumption glucose ≥ 8.9 mmol L^-1^ confirmed a diagnosis of GDM [13]. The presence of specific risk factors (e.g., previous diagnosis of GDM or delivery of a macrosomic infant, member of a high risk population, age > 35, obesity, polycystic ovarian syndrome, acanthosis nigricans, or corticosteroid use) was a clinical indication for the performance of glucose testing in the first trimester. A positive result indicated the presence of “overt diabetes” (usually previously unrecognized or undiagnosed type 2 diabetes) [13,14], which would have been recorded as pre-existing diabetes. A diagnosis of pre-existing diabetes was otherwise made from an assessment of patient history, clinical chart records, and medication records (insulin or oral antihyperglycemic agents). We were unable to discern which pre-existing diabetes cases were type 1 diabetes and which were type 2 diabetes. Descriptions of the other variables included in the analyses are presented in Additional file 1. Because data on breastfeeding were available for just 33% (i.e., not for the entire province) of the pregnant mothers, these data were used only for descriptive purposes and were not included in the regression models. The delivery records included calculation of a composite antepartum risk score, which was a weighted summary measure of pregnancy risk factors (45-item score of pre-pregnancy, previous obstetrical history, problems in current pregnancy and other risk factors). Women that received a score ≥ 7 were considered to have high risk pregnancies. The variables used and the scoring details are available from the APHP [12]. They were used in a recent retrospective study that assessed the association between the Alberta antepartum risk score and perinatal morbidity outcomes [15].

### Population

The three types of Indigenous peoples recognized by Canada are the First Nations, the Métis (mixed heritage), and the Inuit peoples. Each group possesses distinct cultural identities and has distinct governance and healthcare systems. First Nations and Inuit individuals whose Nations have engaged in Treaties are accorded “registered Indian” or Treaty status, which includes a “status card” that confers a number of Treaty rights, including access to healthcare services. The Alberta Health Care Insurance Plan Central Stakeholder Registry file [Ministry of Alberta Health (AH), Surveillance Division] includes an identifier for individuals with registered Indian status. Data obtained from the APHP were sent to AH for matching via the Personal Health Number. First Nations individuals were defined as any Alberta resident registered under the Indian Act of Canada and entitled to Treaty status with the Canadian Government. A woman with a First Nations identifier (i.e., First Nations or Inuit) was classified as “First Nations” if her infant was delivered in Alberta. Other persons recognized by Canada (e.g., the Métis), or self-identified as Indigenous but non-registered (such as First Nations individuals without Treaty status), were included in the general population comparison group because they could not be identified in the administrative database. In Alberta, there are approximately 116,670 First Nations people (19,945 of these are non-registered) and 96,865 Métis people, representing approximately 3.3% and 2.7% of the total population, respectively [16]. Very few Inuit people (approximately 1,600) reside in Alberta. The complete set of de-identified data was returned by AH in STATA statistical software (StataCorp LP, College Station, TX, USA) format.

### Statistical analyses

All analyses were performed using STATA (version 11) and Joinpoint (version 3.5.1, Rockville, Maryland, USA) statistical software. P-values < 0.05 were considered to be statistically significant. The prevalence and mean values for maternal characteristics, pre-existing maternal risk factors, problems with the current pregnancy, maternal outcomes, and newborn outcomes were calculated. Prevalence calculations were completed separately for the following groups: all First Nations pregnancies without diabetes, all non-First Nations pregnancies without diabetes, all First Nations pregnancies with diabetes, all non-First Nations pregnancies with diabetes, all First Nations pregnancies with pre-existing diabetes, and all First Nations pregnancies with GDM. For the prevalence calculations, the denominator was the total number of pregnancies where data on that variable were available for the specific group of interest. The numerator was the total number of pregnancies for which the criteria for the variable of interest was met, for the specific group of interest. Comparisons were by ethnicity (First Nations vs. non-First Nations) among individuals without diabetes and individuals with diabetes. Differences between First Nations women with and without diabetes were also estimated. Finally, comparisons by diabetes type (GDM vs. pre-existing diabetes; type 1 and type 2 diabetes combined) were made among First Nations women with diabetes alone. Chi-square analysis (for categorical variables) and t-tests (for continuous variables) were used for the comparisons.

Our primary focus was to examine trends in, and risk factors for, diabetes in pregnancy. Annual age-adjusted prevalence of GDM and pre-existing diabetes for the entire province by ethnicity were calculated using the direct method. Maternal age distribution data for the total number of pregnancies in 2005 were obtained from Canadian Vital Statistics [17] and were used as the reference population for age-adjustment. Crude prevalence values were also calculated for comparison with results from previous studies. For the longitudinal analyses (i.e., examination of changes in trends over time), the average annual percent change (AAPC) in GDM and pre-existing diabetes prevalence values over time were computed and were compared between ethnic groups. The AAPC provided a summary measure of the trend over a pre-specified fixed interval (2000–2009). Tests of parallelism were then performed to determine whether trends over time differed by ethnic group.

Multivariable logistic regression modeling (purposeful) was used to evaluate the relationships between GDM and potential explanatory variables (i.e., pre-existing maternal risk factors, past obstetrical variables, problems with current pregnancy). The logistic regression analysis was repeated using pre-existing diabetes as the dependent variable. Briefly, independent variables with a p-value < 0.20 in the univariate linear regression analysis were fitted in a multivariable model. Variables with a p-value > 0.05 were removed, and the potential confounding effect of each variable was assessed. The linear assumptions of continuous variables and potential interaction effects were assessed. The Hosmer–Lemeshow test was used to determine the model goodness-of-fit.

## Results

The data received from the APHP indicated that there were 433,445 pregnancies in Alberta between 2000 and 2009. Records where diabetes data were missing (n = 6,387) were not included in the analyses. Of the 427,058 pregnancy records that were examined, 28,306 (6.6%) were from First Nations women. Results for the number of pregnancies where diabetes data were available by year, age group, and ethnicity are presented in Additional file 2. The majority (52.3%) of First Nations pregnancies were among women < 25 years of age. Only 22.5% of non-First Nations women were from this age group.

The results for ethnicity comparisons for selected maternal characteristics, antenatal risk factors, and pregnancy outcomes are presented in Table 1. Between-group comparisons (First Nations vs. non-First Nations) were made among women with and without diabetes during pregnancy. For the majority of the comparisons, First Nations women tended to have more risk factors and poorer outcomes than non-First Nations women. This result suggests that, overall, First Nations women have higher-risk pregnancies. This is indicated most clearly by the elevated prevalence of a composite antepartum risk score of ≥ 7 among First Nations women compared with non-First Nations women. However, First Nations women had less pregnancy-induced hypertension, and fewer labor inductions, NICU admissions, and cesarean sections. Most of these differences by ethnicity persisted when only pregnancies affected by diabetes were compared.

**Table 1:** Selected maternal characteristics, antenatal risk factors, and pregnancy outcomes, by ethnicity and diabetes in pregnancy status, Alberta, Canada, 2000–2009 (n = 427,058)

	**Women without diabetes (n = 407,855)**			**Women with diabetes (n = 19,173)**		
	**First Nations (n = 26,793)**	**Non-First Nations (n = 381,092)**	**P-value**	**First Nations (n = 1,513)**	**Non-First Nations (n = 17,660)**	**P-value**
Maternal characteristics and risks						
Age (years)	24.7 (5.8)	28.7 (5.5)	<0.001	28.9 (6.2)	31.6 (5.3)	<0.001
Rural	51.8 (51.2-52.4)	15.9 (15.8-16.0)	<0.001	52.5 (49.9-55.0)	11.4 (10.9-11.9)	<0.001
Weight ≥ 91 kg	10.8 (10.5-11.2)	8.0 (7.9-8.1)	<0.001	31.7 (29.3-34.1)	18.4 (17.9-19.0)	<0.001
Pre-existing hypertension	0.9 (0.8-1.0)	0.9 (0.9-1.0)	0.367	4.2 (3.3-5.4)	3.3 (3.0-3.5)	0.041
Pregnancy induced hypertension	4.4 (4.2-4.7)	5.7 (5.6-5.7)	<0.001	8.1 (6.7-9.7)	12.2 (11.7-12.7)	0.193
Proteinuria	2.4 (2.2-2.6)	1.9 (1.9-2.0)	<0.001	5.6 (4.5-6.9)	3.9 (3.9-4.2)	0.001
Anemia	2.3 (2.1-2.5)	0.6 (0.5-0.6)	<0.001	1.3 (0.8-2.0)	0.5 (0.4-0.7)	<0.001
Smoker	54.7 (54.1-55.3)	17.0 (16.9-17.2)	<0.001	49.4 (46.8-51.9)	14.3 (13.8-14.8)	<0.001
Alcohol anytime	10.4 (10.0-11.0)	1.7 (1.5-1.8)	<0.001	7.8 (6.2-9.8)	1.0 (0.8-1.1)	<0.001
Drug dependant	6.6 (6.3-6.9)	0.9 (0.8-0.9)	<0.001	3.5 (2.6-4.6)	0.4 (0.3-0.5)	<0.001
Antepartum risk ≥ 7	10.4 (10.1-10.8)	5.1 (5.1-5.2)	<0.001	30.7 (28.4-33.1)	19.8 (19.2-2.4)	<0.001
Pregnancy Outcomes						
Induction of labour	23.8 (23.1-24.3)	27.3 (27.1-27.4)	<0.001	41.7 (39.2-44.2)	40.9 (40.2-41.7)	0.554
Cesarean section	19.1 (18.6-19.5)	25.5 (25.4-25.6)	<0.001	35.4 (33.0-37.8)	39.8 (39.0-40.9)	0.001
Low birth weight	8.0 (7.6-8.3)	7.1 (7.0-7.2)	<0.001	7.2 (6.0-8.6)	8.6 (8.2-9.0)	0.063
High birth weight	16.7 (16.3-17.2)	11.1 (10.9-11.1)	<0.001	29.3 (27.0-31.6)	12.9 (12.4-13.4)	<0.001
Breastfeeding	71.2 (70.5-72.8)	88.3 (88.2-88.6)	<0.001	74.7 (69.3-79.6)	86.3 (85.4-87.1)	<0.001
Preterm	9.2 (8.9-9.6)	8.8 (8.7-8.9)	0.026	17.3 (15.4-19.3)	14.7 (14.2-15.3)	0.008
Stillbirth	1.2 (1.1-1.3)	0.7 (0.6-0.7)	<0.001	2.1 (1.5-2.9)	0.6 (0.5-0.8)	<0.001
Congenital anomaly	1.8 (1.6-2.0)	1.5 (1.4-1.5)	0.001	1.4 (0.8-2.3)	1.7 (1.5-2.1)	0.243
Neonatal intensive care unit admission	8.6 (8.3-9.0)	11.2 (11.1-11.3)	<0.001	16.7 (14.8-15.7)	19.4 (18.8-20.0)	<0.001

Data presented are mean (standard deviation) or percentage (95% confidence intervals).

The results for the within-group comparisons among First Nations women (women without diabetes in pregnancy vs. women with diabetes in pregnancy; women with GDM vs. women with pre-existing diabetes) are presented in Table 2. Compared with women without diabetes, women with diabetes tended to have more pregnancy-related risk factors, including higher composite antepartum risk scores and poorer outcomes. Compared with women with GDM, women with pre-existing diabetes were significantly more likely to have pre-existing risk factors (weight ≥ 91 kg, hypertension), higher composite antepartum risk scores, and adverse infant outcomes (preterm neonate, stillbirth, congenital anomaly, and cesarean section).

**Table 2:** Selected maternal characteristics, antenatal risk factors, and pregnancy outcomes for First Nations pregnancies, by diabetes in pregnancy status and type, Alberta, Canada, 2000–2009 (n = 28,306)

	**Women without diabetes (n = 26,793)**	**Women with diabetes (n = 1,513)**	**P-value**	**Women with pre-existing diabetes (n = 289)**	**Women with GDM (n = 1,224)**	**P-value**
Maternal characteristics and risks						
Age (years)	24.7 (5.8)	28.9 (6.2)	<0.001	29.2 (6.03)	28.8 (6.27)	0.407
Rural	51.8 (51.2-52.4)	52.5 (49.9-55.0)	0.614	55.4 (49.43-61.18)	51.8 (48.96-54.63)	0.275
Weight ≥ 91 kg	10.8 (10.5-11.2)	31.7 (29.3-34.1)	<0.001	37.0 (31.44-42.87)	30.4 (27.85-33.08)	0.030
Pre-existing hypertension	0.9 (0.8-1.0)	4.2 (3.3-5.4)	<0.001	9.3 (6.25-13.30)	3.0 (2.14-4.14)	<0.001
Pregnancy induced hypertension	4.4 (4.2-4.7)	8.1 (6.7-9.7)	<0.001	10.0 (6.82-14.09)	11.3 (9.56-13.18)	0.545
Proteinuria	2.4 (2.2-2.6)	5.6 (4.5-6.9)	<0.001	7.6 (4.83-11.30)	5.2 (3.98-6.54)	0.102
Anemia	2.3 (2.1-2.5)	1.3 (0.8-2.0)	0.013	0.7 (0.08-2.48)	1.5 (0.87-2.31)	0.297
Smoker	54.7 (54.1-55.3)	49.4 (46.8-51.9)	<0.001	48.1 (42.21-54.02)	49.7 (46.83-52.51)	0.630
Alcohol anytime	10.4 (10.0-11.0)	7.8 (6.2-9.8)	0.001	6.9 (3.71-12.33)	7.9 (6.15-10.08)	0.285
Drug dependant	6.6 (6.3-6.9)	3.5 (2.6-4.6)	<0.001	4.9 (2.69-8.05)	3.2 (2.25-4.32)	0.155
Antepartum risk ≥ 7	10.4 (10.1-10.8)	30.7 (28.4-33.1)	<0.001	47.1 (41.19-52.99)	26.9 (24.41-29.46)	<0.001
Pregnancy Outcomes						
Induction of labour	23.8 (23.1-24.3)	41.7 (39.2-44.2)	<0.001	33.6 (28.14-39.33)	43.6 (40.83-46.46)	0.002
Cesarean section	19.1 (18.6-19.5)	35.4 (33.0-37.8)	<0.001	46.7 (40.85-52.65)	32.7 (30.06-35.39)	<0.001
Low birth weight	8.0 (7.6-8.3)	7.2 (6.0-8.6)	0.308	9.0 (5.96-12.90)	6.8 (5.45-8.35)	0.193
High birth weight	16.7 (16.3-17.2)	29.3 (27.0-31.6)	<0.001	26.3 (21.32-31.77)	30.0 (27.39-32.61)	0.220
Breastfeeding	71.2 (70.5-72.8)	74.7 (69.3-79.6)	0.266	77.5 (61.55-89.16)	74.2 (68.34-79.49)	0.656
Preterm	9.2 (8.9-9.6)	17.3 (15.4-19.3)	<0.001	22.8 (18.13-28.12)	16.0 (13.96-18.15)	0.005
Stillbirth	1.2 (1.1-1.3)	2.1 (1.5-2.9)	0.002	3.8 (1.92-6.71)	1.7 (1.07-2.61)	0.027
Congenital anomaly	1.8 (1.6-2.0)	1.4 (0.8-2.3)	0.437	3.1 (1.36-6.06)	0.9 (0.43-1.79)	0.032
Neonatal intensive care unit admission	8.6 (8.3-9.0)	16.7 (14.8-15.7)	<0.001	19.0 (14.62-24.07)	16.1 (14.08-18.31)	0.326

Data presented are mean (standard deviation) or percentage (95% confidence intervals).

The overall crude and age-adjusted GDM prevalence values were significantly elevated for First Nations women compared with non-First Nations women. The First Nations to non-First Nations rate ratio was higher after age-adjustment (Table 3). Similarly, First Nations women had a higher prevalence of crude and age-adjusted pre-existing diabetes than non-First Nations women for all of the years included in the analysis (Table 3). Age-adjustment also resulted in a greater rate ratio between ethnicities for this comparison.

**Table 3:** Crude and age-adjusted prevalence of GDM and pre-existing diabetes for all years (n = 427,058) and for the most recent year of data (2009; n = 51,231), by ethnicity, Alberta, Canada

	**First Nations**	**Non-First Nations**	Rate ratio^*^	**P-value**
Crude				
GDM (all years)	4.3 (4.3-4.4)	3.8 (3.8-3.9)	1.1	<0.001
GDM (2009)	4.9 (4.2-4.6)	4.8 (4.6-5.0)	1.0	0.861
Pre-existing diabetes (all years)	1.0 (1.0-1.1)	0.6 (0.6-0.7)	1.7	<0.001
Pre-existing diabetes (2009)	1.1 (0.8-1.5)	0.7 (0.6-0.8)	1.6	0.006
Age-adjusted				
GDM (all years)	6.1 (6.0-6.1)	3.8 (3.7-3.9)	1.6	<0.001
GDM (2009)	6.9 (6.8-6.9)	4.6 (4.6-4.7)	1.5	<0.001
Pre-existing diabetes (all years)	1.5 (1.4-1.5)	0.6 (0.6-0.6)	2.5	<0.001
Pre-existing diabetes (2009)	1.4 (1.3-1.4)	0.7 (0.6-0.7)	2.0	<0.001

Data presented are percentage (95% confidence interval).

GDM, gestational diabetes mellitus.

^*^First Nations-to-non-First Nations.

**Figure 1:**
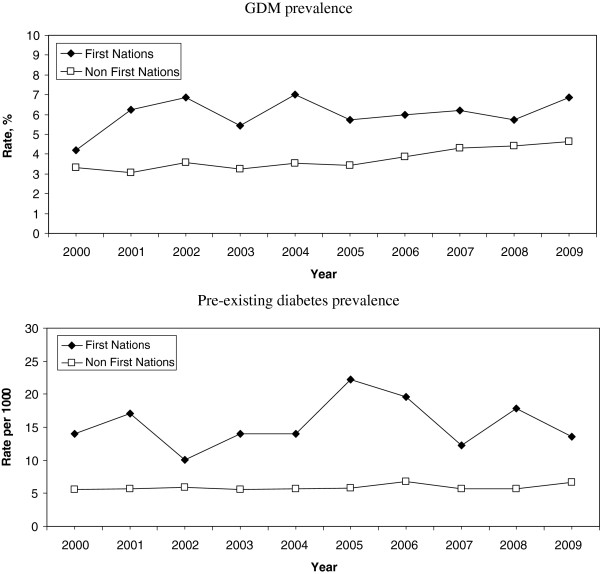
**Age-adjusted prevalence of diabetes in pregnancy over time, by ethnic group.** Top = GDM prevalence, bottom = pre-existing diabetes prevalence.

During 2000 to 2009, age-adjusted GDM prevalence increased significantly only among non-First Nations women (Figure 1; p = 0.02 for the between-ethnicity difference in AAPC). The AAPC for GDM was 1.51 [95% confidence interval (CI): -2.04–5.20; p = 0.20] among First Nations women and 4.48 (95% CI: 2.88–6.11; p = 0.01) among non-First Nations women. Prevalence of pre-existing diabetes did not increase over time among either First Nations or non-First Nations women, and there were no between-group differences (Figure 1; p = 0.95 for the difference in AAPC between ethnicities). The AAPCs for pre-existing diabetes were 1.55 (95% CI: -6.68–8.19; p = 0.32) among First Nations women and 1.35 (95% CI: -0.38–3.12; p = 0.18) among non-First Nations women.

**Figure 2:**
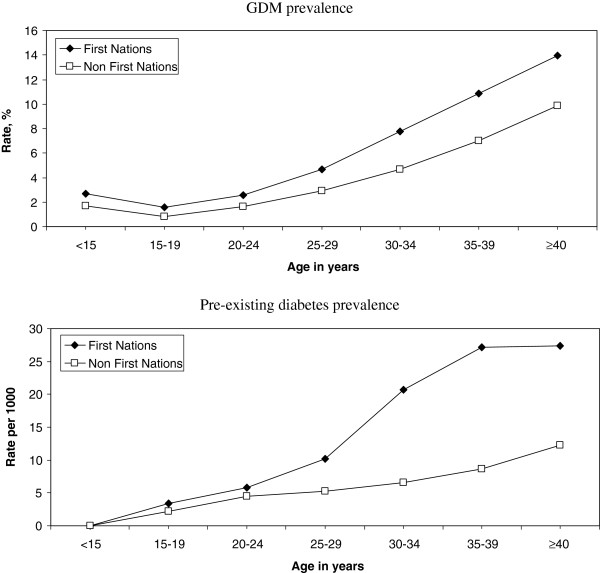
**Age-specific prevalence of diabetes for all years in pregnancy, by ethnic and age groups.** Top = GDM prevalence, bottom = pre-existing diabetes prevalence.

Figure 2 presents the results for prevalence of age-specific diabetes in pregnancy by ethnicity over the entire time period. GDM prevalence was lowest for both groups among women aged 15–19 years and increased with increasing age. Pre-existing diabetes prevalence values were considerably higher among First Nations women aged 30–34 years and 35–39 years, which is consistent with the presence of early onset type 2 diabetes in these women.

The results for the final adjusted logistic regression models and estimated odds ratios (ORs) are presented in Table 4. Among all women, the factors significantly associated with an increased risk of GDM included First Nations ethnicity, age ≥ 35 years, weight ≥ 91 kg, pre-existing hypertension, previous stillbirth, previous cesarean section, and proteinuria. Age ≤ 17 years, rural residence, smoking, and drug dependence were associated with a lower risk of GDM. Among First Nations women, there were significant associations with increased GDM risk for age ≥ 35 years, weight ≥ 91 kg, previous stillbirth, previous cesarean section, previous birthing of an infant that was large for gestational age (LGA), and proteinuria. Women ≤ 17 years and with drug dependence had a lower risk of GDM.

**Table 4:** Multivariate predictors of GDM and pre-existing diabetes among all Albertan women and all First Nations women, by ethnicity

	**All women**				**First Nations**			
	**(n = 427,058)**				**(n = 28,306)**			
**Variable**	**OR (95% CI) for GDM**	**P-value**	**OR (95% CI) for pre-existing diabetes**	**P-value**	**OR (95% CI) for GDM**	**P-value**	**OR (95% CI) for pre-existing diabetes**	**P-value**
First Nations ethnicity	1.47 (1.38-1.57)	<0.001	1.73 (1.52-1.96)	<0.001	–	–	–	–
Rural residence	0.69 (0.66-0.73)	<0.001	–	–	–	–	–	–
Age ≤ 17^*^	0.35 (0.28-0.45)	<0.001	0.29 (0.16-0.52)	<0.001	0.48 (0.34-0.67)	<0.001	0.42 (0.18-0.94)	0.035
Age ≥ 35^*^	2.34 (2.26-2.42)	<0.001	1.57 (1.43-1.72)	<0.001	2.81 (2.41-3.27)	<0.001	2.23 (1.64-3.02)	<0.001
Weight ≥ 91 kg	2.51 (2.40-2.61)	<0.001	2.31 (2.10-2.54)	<0.001	2.93 (2.56-3.33)	<0.001	3.25 (2.52-4.18)	<0.001
Hypertension	1.63 (1.45-1.84)	<0.001	4.45 (3.82-5.43)	<0.001	–	–	5.09 (3.03-8.58)	<0.001
Previous stillbirth	1.96 (1.75-2.18)	<0.001	2.56 (2.07-3.18)	<0.001	1.76 (1.30-2.38)	<0.001	3.05 (1.93-4.81)	<0.001
Previous abortion	–	–	1.42 (1.23-1.64)	<0.001	–	–	1.58 (1.11-2.25)	0.012
Previous cesarean section	1.37 (1.32-1.43)	<0.001	1.76 (1.61-1.94)	<0.001	1.40 (1.20-1.63)	<0.001	1.88 (1.41-2.47)	<0.001
Previous small for gestational age infant	–	–	0.53 (0.29-0.96)	0.038	–	–	–	–
Previous large for gestational age infant	–	–	2.79 (2.31-3.39)	<0.001	2.58 (1.99-3.35)	<0.001	3.23 (2.10-4.97)	<0.001
Proteinuria	1.61 (1.48-1.76)	<0.001	2.62 (2.23-3.08)	<0.001	1.85 (1.40-2.44)	<0.001	2.05 (1.26-3.32)	0.04
Smoker	0.90 (0.86-0.94)	<0.001	–	–	–	–	–	–
Drug dependant	0.51 (0.41-0.63)	<0.001	–	–	0.54 (0.39-0.74)	<0.001	–	–
Alcohol anytime	–	–	0.68 (0.50-0.93)	0.016	–	–	–	–

Data presented are OR, odds ratios (95% confidence intervals);

GDM, gestational diabetes mellitus.

^*^Compared with age 18-34.

Among all women, factors significantly associated with an increased risk of pre-existing diabetes included First Nations ethnicity, age ≥ 35 years, weight ≥ 91 kg, pre-existing hypertension, previous stillbirth, previous abortion, previous cesarean section, previous LGA infant, and proteinuria. Age ≤ 17 years, having a previous small for gestational age infant and alcohol consumption during pregnancy were associated with a lower risk of pre-existing diabetes. Among First Nations women, there were significant associations with increased pre-existing diabetes risk for the variables age ≥ 35 years, weight ≥ 91 kg, previous stillbirth, previous abortion, previous cesarean section, previous LGA infant, and proteinuria. Being at an age ≤ 17 years conferred a lower risk of pre-existing diabetes.

## Discussion

Our large sample size over a 10-year period allowed for analyses that indicated that having diabetes in pregnancy (particularly pre-existing diabetes) compounds already increased risks of adverse pregnancy outcomes for First Nations women. The results of our study are unique in that they are the first results that describe large-scale trends over time for both pre-existing diabetes and GDM in a First Nations population. Although GDM prevalence was disproportionately higher among First Nations women, we found that prevalence is growing more rapidly among non-First Nations women. First Nations women also suffer a greater than 2-fold higher prevalence of pre-existing diabetes, which is likely contributed to by early onset of type 2 diabetes in the prime child-bearing years (i.e., twenties and thirties) [6]. However, the prevalence of pre-existing diabetes in pregnancy was generally stable over time amongst both groups. An additional novel finding was that the epidemiological profile of diabetes in pregnancy in Alberta does not appear to be as severe in comparison with other provinces. Our study also provides new information about previously unexplored maternal characteristics and antenatal risk factors, and their associations with diabetes in pregnancy.

This study is the first to calculate age-adjusted GDM and pre-existing diabetes prevalence among a First Nations population. Because advancing maternal age is a risk factor for GDM, and the First Nations population consists mostly of younger age groups [3,18], the results presented in previous reports may underestimate the differences between First Nations and general populations. Being of First Nations descent was independently associated with both GDM and pre-existing diabetes in pregnancy. Finally, in addition to risk factors identified in previous studies [19-21], the results of this study indicate that previous stillbirth, previous cesarean section, previous abortion (spontaneous or therapeutic), previous LGA infant, and the presence of proteinuria are also independently associated with GDM and pre-existing diabetes in pregnancy in Indigenous women.

Values for several components of the total antepartum risk score are higher in other First Nations populations. These components include previous preterm birth [22], smoking during pregnancy [23], fetal exposure to illicit drugs [24], and stillbirth [25]. In the current study, pregnancy risk factors that showed the highest disparity between First Nations and non-First Nations women included smoking anytime during pregnancy (3.2-fold), the presence of anemia (3.8-fold), alcohol consumption any time during pregnancy (6.1-fold), and drug dependency (7.3-fold). It is likely that these factors contribute to the observed poorer outcomes among First Nations infants, and interventions are clearly required to mitigate these risks. The ethnic inequalities persisted in women who only had diabetes in pregnancy. To our knowledge, this was the first study to find adverse effects by First Nations status among women with diabetes, associated with high pre-existing weight, pre-existing hypertension, proteinuria, anemia, stillbirth, smoking, alcohol consumption, and drug dependency. All of these factors were significantly more common among First Nations women. This study is also the first to compare risk factors and pregnancy outcomes by diabetes status among First Nations women only. Having diabetes (especially pre-existing) clearly increased the risk of several adverse pregnancy risk factors and outcomes, similar to previously reported results for non-Indigenous women [26].

This study confirmed the results of previous studies that found a higher prevalence of GDM among First Nations women [9,19,20,27]. However, the epidemiological profile in Alberta did not seem to be as severe as in other provinces. The crude First Nations GDM prevalence in Alberta (4.3%) was lower compared with First Nations populations in other parts of Canada, such as Manitoba (6.9%), Quebec (8.5%), and northwestern Ontario (8.4%) [20,21,27]. The First Nations to non-First Nations crude rate ratio of 1.1 for GDM prevalence was also lower in Alberta compared with the 1.8 and 2.9 ratios reported for women in the provinces of Saskatchewan and Manitoba, respectively [19,20]. Similarly, we have recently found that rate ratios for overall diabetes incidence and prevalence are also lower in Alberta compared with other provinces [7]. The results of our study cannot explain provincial differences, but we speculate they may be related to socio-economic differences, and/or a combination of provincial and community-based programming targeting awareness such as the provincial Alberta Diabetes Strategy, the federally funded Aboriginal Diabetes Initiative, or changes in clinical practice. Future studies are needed to uncover reasons for regional variations, and should include age-adjustments for more informative comparisons across ethnic groups.

GDM prevalence is increasing in many populations worldwide [28], and this pattern is also present among non-First Nations Albertan women. The increasing age of pregnant non-First Nations women and an influx of minority immigrants likely have contributed to this increase in prevalence [20,29]. The situation does not seem as clear among Indigenous populations, because prevalence is not increasing significantly in Alberta First Nations women. This is consistent with our article on overall diabetes prevalence in Alberta [7], which reported that overall diabetes prevalence is increasing more rapidly among the non-First Nations population. One US study found that GDM prevalence among American Indian women increased from 3.1% to 4.1% during 1989–2000 [30]. However, studies among Aborigines in Australia have found that prevalence values are stable or even decreasing over time [31,32].

Liu et al [33] reported a higher pre-existing diabetes prevalence in Ontario First Nations women (3.9%) compared with their non-First Nations counterparts (1.8%). This result is consistent with our results and with the increased numbers of young women of child-bearing age with type 2 diabetes that we and others have documented in First Nations populations [6,7]. As with GDM, pre-existing diabetes prevalence is lower among Alberta First Nations (1.0% crude) compared with Ontario First Nations women.

To our knowledge, this study is the first to describe trends in pre-existing diabetes in pregnancy over time in an Indigenous population. The stability of prevalence in the First Nations and non-First Nations populations is surprising and encouraging, because the prevalence of pre-existing diabetes in pregnancy is increasing worldwide [34] and overall diabetes prevalence appears to be increasing in both the First Nations and non-First Nations populations [7].

There were several limitations of this study. The results cannot be generalized to non-registered First Nations individuals or to Métis individuals. These individuals could not be identified and were included in the non-First Nations population group. Also, the completeness of coverage of the First Nations identification could not be determined, so the observed ethnic (First Nations vs. non-First Nations) disparities could have been underestimated. Screening for diabetes in early pregnancy is often not performed (personal observation, E.L.T.) even though it is recommended for women with multiple risk factors [13]. Therefore, it is also likely that some First Nations women with pre-existing diabetes were not diagnosed, and were later classified as having GDM. This error would underestimate the magnitude of the findings of worse outcomes with pre-existing diabetes than with GDM. Whether the recorded pre-existing diabetes cases were type 1 or type 2 diabetes could not be discerned from the administrative data. Clinical experience suggests that most diabetes in young First Nations women is type 2 diabetes, but the prevalence of type 2 diabetes amongst non-First Nations populations (as we defined them) is also increasing. We believe that this change is due to obesity, immigration, and the inclusion of non-registered Indigenous persons. Further research is needed to determine the specific contributions of type 1 and type 2 diabetes, but glucose control is equally important to perinatal outcomes regardless the type of pre-existing diabetes. The degree to which diabetes was successfully managed (glucose control, hemoglobin A1c) also could not be determined for the study population. Finally, the contribution of other potential contextual predictors (e.g., healthcare access, lifestyle, overweight/obesity, social environment, income) to the logistic regression models could not be assessed.

## Conclusion

First Nations women suffer a higher prevalence of diabetes in pregnancy and more adverse pregnancy outcomes, but the prevalence in Alberta does not appear to be as high as it is in other provinces. Future studies are needed to uncover reasons for regional variations and should use age-adjustment for more informative comparisons across ethnic groups. Future and ongoing monitoring of diabetes prevalence in pregnancy, information on pregnancy outcomes, and epidemiological data on diabetes in pregnancy among other Indigenous populations (Métis, Inuit, non-registered Indigenous women) will be necessary. More complex and inclusive studies should be used to assess the contribution of other potential contextual predictors (e.g., healthcare access, lifestyle, social environment, income, medical management). Our findings should allow government and healthcare organizations to develop policy and plan healthcare delivery, and to evaluate efforts and assess their cost-effectiveness, so that perinatal care in this population can be improved.

## Abbreviations

AAPC: Average annual percent change; AH: Alberta health; APHP: Alberta perinatal health program; CI: Confidence interval; GDM: Gestational diabetes mellitus; LGA: Large for gestational age; NICU: Neonatal intensive care unit; OR: Odds ratio.

## Competing interests

The authors declare that they have no competing interests.

## Authors’ contributions

RTO contributed to the study design, obtained the data, conducted statistical analyses, and wrote the manuscript. MK, DWM, and MJM contributed to the study design and the discussion, and reviewed and edited the manuscript. ELT was the principal investigator, contributed to the study design and the discussion, and reviewed and edited the manuscript. All authors read and approved the final manuscript.

## Pre-publication history

The pre-publication history for this paper can be accessed here:

http://www.biomedcentral.com/1471-2393/14/136/prepub

## Supplementary Material

Additional file 1 Description of additional variables that were included. Click here for file

Additional file 2 Number of pregnancies with diabetes data, by age group and ethnicity. Click here for file
