# Supplementary material for: Diabetes in pregnancy among First Nations women in Alberta, Canada: a retrospective analysis
Source: BMC Pregnancy Childbirth. 2014 Apr 10;14:136. doi: 10.1186/1471-2393-14-136 (PMC4021202; doi:10.1186/1471-2393-14-136)
Supplement: Additional file 1 — Description of additional variables that were included. [file 1471-2393-14-136-S1.doc]

Additional File 1. Description of additional variables that were included.

| Maternal characteristics and risks |  |
| --- | --- |
| Age  (n = 426,335) | Maternal age in years at delivery |
| Weight ≥ 91 kg  (n = 426,913) | Maternal pregestational weight ≥ 91 kg |
| Pre-existing hypertension  (n = 427,058) | Pregestational blood pressure ≥ 140/90 mmHg and/or taking antihypertensive drugs |
| Diabetes retinopathy  (n = 427,058) | Pregestational diagnosis of diabetic retinopathy |
| Previous preterm  (n = 262,459) | History of infants born between 20 and < 37 weeks excluding current (southern Alberta did not collect until 2007) |
| Previous stillbirth  (n = 427,058) | History of birth (after ≥ 20 weeks or attaining a weight of ≥ 500g) of a fetus that has died in utero |
| Previous abortion  (n = 427,058) | History of abortion between 12 to 20 weeks and < 500 g weight (therapeutic and spontaneous) |
| Previous cesarean section  (n = 427,058) | History of cesarean section |
| Previous small for gestational age  ( n = 427,058) | History of infant birth weight < 5^th^ percentile for gestational age |
| Previous large for gestational age  ( n = 427,058) | History of infant birth weight > 95^th^ percentile for gestational age |
| Pregnancy induced hypertension  (n = 427,058) | Diagnosis of gestational hypertension (blood pressure ≥ 140/90 mmHg) during current pregnancy |
| Proteinuria  (n = 427,058) | Proteinuria (≥ 0.3g/d 24 hour urine collection or ≥ 30 mg/mmol/ urinary creatinine in a random urine sample) with current pregnancy |
| Anemia  (n = 427,058) | Anemia (hemoglobin < 100 g/L) with current pregnancy |
| Smoker  (n = 427,058) | Smoker anytime during current pregnancy |
| Alcohol ≥ 1 drink per day  (n = 414,404) | Alcohol ≥ 1 drink per day throughout current pregnancy |
| Alcohol ≥ 3 drinks ever  (n = 414,404) | Alcohol ≥ 3 drinks on any one occasion during current pregnancy |
| Drug dependant  (n = 414,549) | Inappropriate/excessive use of a substance that may adversely affect the pregnancy or newborn in current pregnancy |
| Antepartum risk score  (n = 426,951) | Sum of the weighted values for antepartum risk assessment factors from the delivery record (45-item score of pre-pregnancy, past obstetrical history, problems in current pregnancy and other risk factors; see reference 12) |
| Pregnancy Outcomes |  |
| Induction of labour  (n = 427,018) | Woman had induced labour in current pregnancy |
| Birth weight  (n = 426,235) | First weight of newborn after birth, in grams |
| Low birth weight  (n = 426,235) | First weight of newborn after birth ≤ 2500 g |
| High birth weight  (n = 426,235) | First weight of newborn after birth ≥ 4000 g |
| Breastfeeding  (n = 141,985) | Infant breastfed either after delivery or upon on discharge (data not available province-wide) |
| Preterm  (n = 426,462) | Infant born to this woman between 20 and < 37 completed weeks gestation in current birth |
| Stillbirth  (n = 427,058) | Birth (after ≥ 20 weeks or attaining a weight of ≥ 500 g) of a fetus that has died in utero |
| Neonatal intensive care unit admission (n = 417,751) | Baby admitted to NICU as the clinical care that is required is beyond that of a healthy newborn |
| Major congenital anomaly  (n = 414,549) | Presence of major fetal anomaly at birth |
| Cesarean section  (n = 427,058) | Cesarean section delivery method |
