# Supplementary material for: Diabetes in pregnancy among First Nations women in Alberta, Canada: a retrospective analysis
Source: BMC Pregnancy Childbirth. 2014 Apr 10;14:136. doi: 10.1186/1471-2393-14-136 (PMC4021202; doi:10.1186/1471-2393-14-136)
Supplement: Additional file 2 — Number of pregnancies with diabetes data, by age group and ethnicity. [file 1471-2393-14-136-S2.doc]

|  | Year | | | | | | | | | |  |
| --- | --- | --- | --- | --- | --- | --- | --- | --- | --- | --- | --- |
| Age and ethnicity | 2000 | 2001 | 2002 | 2003 | 2004 | 2005 | 2006 | 2007 | 2008 | 2009 | % of total pregnancies |
| < 15 |  |  |  |  |  |  |  |  |  |  |  |
| First Nations | 7 | 5 | 5 | 9 | 8 | 8 | 5 | 10 | 6 | 12 | 0.3% |
| Non-First Nations | 9 | 14 | 13 | 6 | 12 | 6 | 18 | 18 | 14 | 8 | 0.0% |
| 15-19 |  |  |  |  |  |  |  |  |  |  |  |
| First Nations | 516 | 526 | 510 | 497 | 498 | 570 | 564 | 585 | 642 | 677 | 19.8% |
| Non-First Nations | 1 863 | 1 733 | 1 698 | 1 600 | 1 567 | 1 544 | 1 704 | 1 849 | 1 797 | 1 685 | 4.3% |
| 20-24 |  |  |  |  |  |  |  |  |  |  |  |
| First Nations | 800 | 751 | 847 | 900 | 912 | 924 | 965 | 985 | 1 035 | 1 060 | 32.5% |
| Non-First Nations | 6 473 | 6 663 | 6 889 | 7 087 | 6 913 | 7 010 | 7 582 | 8 027 | 7 993 | 7 781 | 18.2% |
| 25-29 |  |  |  |  |  |  |  |  |  |  |  |
| First Nations | 531 | 596 | 660 | 654 | 635 | 651 | 740 | 820 | 882 | 878 | 24.9% |
| Non-First Nations | 10 500 | 10 681 | 11 089 | 11 638 | 11 977 | 12 445 | 13 203 | 14 521 | 15 261 | 15 765 | 31.9% |
| 30-34 |  |  |  |  |  |  |  |  |  |  |  |
| First Nations | 346 | 358 | 374 | 390 | 414 | 390 | 439 | 469 | 507 | 516 | 14.9% |
| Non-First Nations | 9 380 | 10 031 | 10 519 | 11 029 | 11 235 | 11 606 | 12 451 | 13 349 | 14 197 | 14 716 | 29.8% |
| 35-40 |  |  |  |  |  |  |  |  |  |  |  |
| First Nations | 157 | 151 | 142 | 168 | 168 | 163 | 182 | 203 | 214 | 225 | 6.3% |
| Non-First Nations | 4 401 | 4 514 | 4 620 | 4 801 | 4 818 | 5 096 | 5 525 | 6 091 | 6 440 | 6 552 | 13.3% |
| ≥40 |  |  |  |  |  |  |  |  |  |  |  |
| First Nations | 25 | 43 | 26 | 39 | 31 | 36 | 34 | 35 | 44 | 52 | 1.3% |
| Non-First Nations | 758 | 794 | 816 | 979 | 972 | 1 028 | 1 051 | 1 181 | 1 203 | 1 299 | 2.5% |
| Total |  |  |  |  |  |  |  |  |  |  |  |
| First Nations | 2 399 | 2 457 | 2 566 | 2 662 | 2 672 | 2 743 | 2 935 | 3 121 | 3 331 | 3 420 | 28 306 |
| Non-First Nations | 33 527 | 34 581 | 35 688 | 37 192 | 37 539 | 38 775 | 41 588 | 45 109 | 46 942 | 47 811 | 398 752 |

Additional File 2. Number of pregnancies with diabetes data, by age group and ethnicity
